# Huafengdan Inhibits Glioblastoma Cell Growth and Mobility by Acting on PLAU and CAV1 Targets

**DOI:** 10.3390/ph18030428

**Published:** 2025-03-18

**Authors:** Dengxiao Lin, Wenfeng Yu, Jia Yu, Sha Cheng, Yu Song, Xiaoqing Wan, Yingjiang Xu, Heng Luo, Baofei Sun

**Affiliations:** 1 Key Laboratory of Human Brain Bank for Functions and Diseases, Department of Education of Guizhou Province, College of Basic Medical, Guizhou Medical University, Guiyang 561113, China; lindengxiao@163.com (D.L.); wenfengyu@gmc.edu.cn (W.Y.); 2 State Key Laboratory of Discovery and Utilization of Functional Components in Traditional Chinese Medicine, Guizhou Medical University, Guiyang 550014, China; yujia@gzcnp.cn (J.Y.); chengsha@gzcnp.cn (S.C.); 3 Natural Products Research Center of Guizhou Province, Guizhou Medical University, Guiyang 550014, China; 4 Guizhou Wansheng Pharmaceutical Co., Ltd., Zunyi 563005, China; 18323539745@163.com (Y.S.); 13985198830@163.com (X.W.); 18781726591@163.com (Y.X.)

**Keywords:** Huafengdan, glioblastoma, PLAU, CAV1, network pharmacology, ethnomedicine

## Abstract

**Background**: Glioblastoma (GBM) is considered a clinically refractory malignant tumor due to its high recurrence and malignancy, invasiveness, and poor prognosis. The ethnomedicine Huafengdan (HFD) is prepared using several Chinese herbs by a complex fermentation process that has a long history. Previous studies have reported the inhibitory effect of HFD on GBM both in vitro and in vivo; however, its mechanism of action is unclear. **Methods**: The inhibitory effects of HFD on the growth, migration, and invasion of GBM cells were determined using the MTT assay, EdU assay, Transwell assay, flow cytometry, and Western blotting. A subcutaneous graft tumor model of nude BALB/c mice was established using U87 cells, and the in vivo activity and toxicity of HFD were evaluated using immunohistochemical staining and hematoxylin and eosin staining. Network pharmacology, bioinformatics, and transcriptomics were used to screen the targets and related signaling pathways of HFD in GBM and were validated using qPCR, CETSA, and Western blotting. **Results**: HFD inhibited the proliferation, invasion, and migration of GBM cells and induced S-phase block and apoptosis in GBM cells. It inhibited the in vivo growth of GBM cells without obvious toxicity. Mechanistic studies showed that the inhibition of GBM cell growth, migration, and invasion by HFD involved the key targets PLAU and CAV1. Its associated signaling pathways were the PI3K/Akt signaling pathway and cell cycle signaling pathway. **Conclusions**: Our findings confirm the novel function of HFD in inhibiting GBM cell growth in vitro and in vivo and highlight its potential in treating GBM.

## 1. Introduction

Gliomas are malignant tumors located in the central nervous system and are the most common type of primary intracranial tumors, accounting for 81% of all intracranial malignancies [1]. Although gliomas are relatively rare, they are associated with high mortality and recurrence rates. The annual incidence of gliomas in China is approximately 4–8/100,000 and has a five-year mortality rate that is second to pancreatic and lung cancers [2]. Gliomas can be classified into four grades (I–IV) based on the classification of central nervous system tumors (WHO and CBTRUS). Glioblastoma (GBM) is a grade-IV tumor which accounts for half of all gliomas [3] and is characterized by high recurrence, high malignancy, high aggressiveness, and poor prognosis. The main clinical treatments for GBM currently include the surgical removal of lesions and moderate doses of radiation therapy and temozolomide (TMZ) adjuvant chemotherapy [4]. Although the survival rate of some patients has improved slightly after treatment with standard regimens, the overall prognosis remains poor, with only about 30% of patients achieving two-year survival and fewer than 10% of patients surviving for more than three years [5,6]. The first-line chemotherapeutic drug TMZ mainly induces DNA methylation in glioma cells to exert a therapeutic effect; however, the outcome is often affected by various factors. GBM cells are susceptible to sustained resistance to TMZ, often resulting in poor prognosis of patients [7]. Therefore, there is an urgent need to identify novel drugs with low drug resistance and less toxic side effects to treat GBM.

Traditional Chinese medicine (TCM) has been used to treat brain tumors for thousands of years [8,9]. TCM preparations are composed of complex and diverse components, which act via multiple pathways and multiple targets. This synergistic effect of multiple components can simultaneously enhance efficacy and reduce the occurrence of drug resistance [10]. As TCMs are composed of multiple active ingredients, these preparations can act on multiple drug targets, and this multitargeting effect provides a more comprehensive means of the regulation and treatment of intractable diseases, thereby providing better choices for the treatment of several complex diseases [11]. Liao’s Huafengdan (HFD) is one of the “Four Great Ancient Prescriptions” in China and is composed of more than 20 medicinal ingredients including Mother Medicine, *Perilla frutescens*, Arisaematis Rhizoma, *Atractylodes lancea* (Thunb.) DC., Crotonis Fructus, *Moschus moschiferus*, *Borneolum syntheticum*, *Gastrodia elata*, Schizonepetae Herba, and *Santalum album* L. Mother Medicine is prepared by mixing, fermenting, and air-drying traditional Chinese medicines such as Typhonii Rhizoma, *Arum ternatum* Thunb., Aconiti Radix, and Curcumae Radix in specific proportions [12]. Clinically, HFD has been used for more than 300 years and is mainly used to treat stroke, epilepsy, acute cerebral infarction, facial nerve paralysis, cerebral atrophy, and other brain diseases with remarkable efficacy [13,14,15,16].

Our research group has previously reported the significant activity of HFD against leukemia, prostate cancer, gastric cancer, and glioma based on screening for antitumor activity. Based on this series of screening, the effectiveness of HFD in treating GBM was determined based on in vivo and in vitro studies, network pharmacology, bioinformatics, and transcriptomics. Accordingly, the molecular targets and related signaling pathways of HFD in regulating the growth, migration, and invasion of GBM cells were analyzed, and the regulatory effects of these targets and signaling pathways were validated using modern molecular biology techniques to elucidate the molecular mechanism of HFD in treating GBM and provide novel ideas to identify drugs to treat GBM.

## 2. Results

### 2.1. HFD Inhibited the Growth of GBM Cells In Vitro

**Figure 1:**
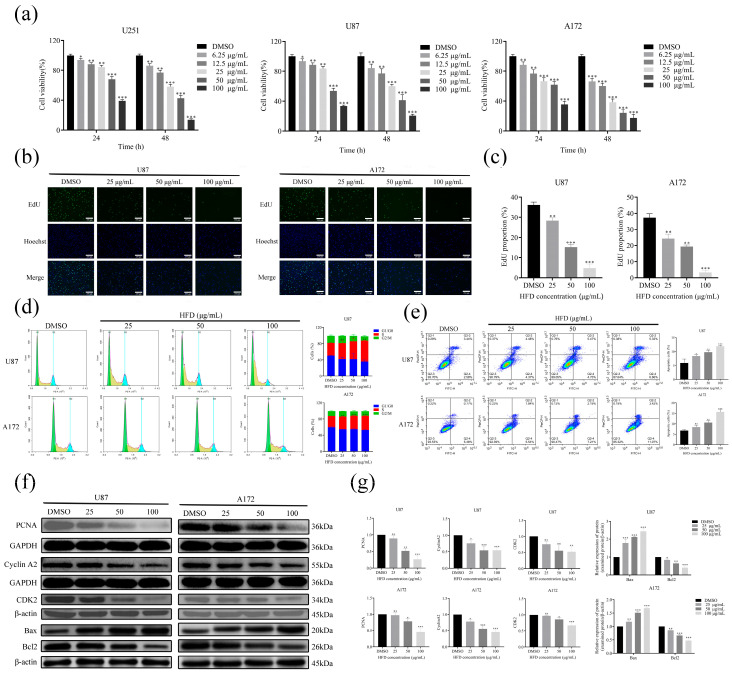
HFD inhibits proliferation and induces S-phase block and apoptosis in GBM cells in vitro. (**a**) Changes in cell viability after treatment with different concentrations of HFD (0, 6.25, 12.5, 25, 50, and 100 μg/mL) for 24 h and 48 h were determined using the MTT assay. (**b**,**c**) Percentage of EdU-positive cells after treatment of U87 and A172 cells with different concentrations of HFD (0, 25, 50, and 100 μg/mL) for 24 h. (**d**) Cell cycle distribution of each group of cells after treatment with different concentrations of HFD was determined using flow cytometry. (**e**) Flow cytometry to determine apoptosis in U87 and A172 cells treated with different concentrations of HFD. (**f**,**g**) Western blotting to determine the expression of PCNA, cyclin A2, CDK2, Bax, and Bcl2 proteins. Data are presented as mean ± SD, n = 3, * *p* < 0.05; ** *p* < 0.01; *** *p* < 0.001, compared with the DMSO group. GBM, glioblastoma; HFD, Huafengdan; SD, standard deviation.

The MTT assay was conducted to evaluate the effects of various concentrations of HFD on the viability of GBM cells at 24 and 48 h. The results demonstrated that HFD significantly inhibited the proliferation of GBM cells compared to the control group (DMSO) (Figure 1a). At 24 h, the IC50 values of HFD for U251, U87, and A172 cells were determined to be 81.423 μg/mL, 61.234 μg/mL, and 60.385 μg/mL, respectively. Based on these findings, U87 and A172 cells were selected for subsequent experiments, and HFD concentrations of 25 μg/mL, 50 μg/mL, and 100 μg/mL were chosen for further investigation. Subsequently, the effect of HFD on GBM cell proliferation was determined using EdU staining, and HFD was found to significantly inhibit DNA synthesis in GBM cells compared with the control (DMSO) (Figure 1b,c). The effects of HFD on the cell cycle and apoptosis in GBM cells were determined using flow cytometry, and HFD was found to block GBM cells in the S-phase (Figure 1d) and promote apoptosis (Figure 1e) compared with the control (DMSO). In addition, the effects of HFD on the expression of proliferation-related protein PCNA, S-phase–related proteins cyclin A2 and CDK2, and apoptosis-related proteins Bax and Bcl2 in GBM cells were determined using Western blotting. HFD downregulated the expression of PCNA, cyclin A2, CDK2, and Bcl2 proteins and upregulated Bax protein expression (Figure 1f,g) compared with the control (DMSO). In summary, HFD inhibited the growth of GBM cells in vitro.

### 2.2. HFD Inhibited the Migration and Invasion Ability of GBM Cells

**Figure 2:**
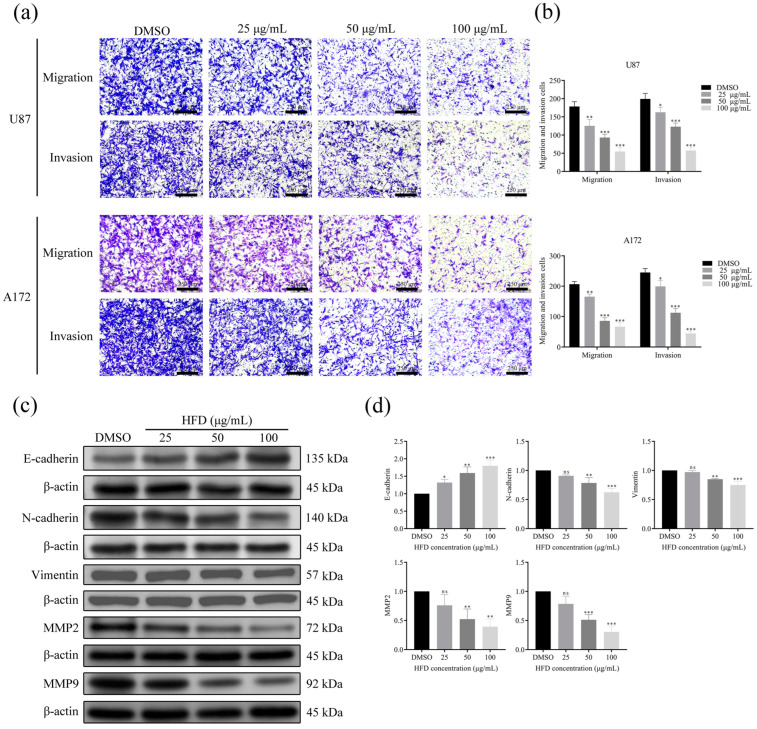
HFD inhibits the migration and invasion of GBM cells. (**a**,**b**) Transwell assay to determine changes in the migration and invasion abilities of U87 and A172 cells after treatment with different HFD concentrations (0, 25, 50, and 100 μg/mL). (**c**,**d**) Western blotting to determine the expression of N-cadherin, E-cadherin, Vimentin, MMP2, and MMP9 proteins in U87 cells treated with different HFD concentrations. Data are presented as mean ± SD, n = 3, * *p* < 0.05; ** *p* < 0.01; *** *p* < 0.001, compared with the DMSO group. MMP, matrix metalloproteinase.

The effect of HFD on GBM cell migration and invasion was evaluated using the Transwell assay. The number of U87 and A172 cells that migrated and invaded decreased with increasing concentrations of HFD compared with the control (DMSO) (Figure 2a,b). In addition, results from Western blotting indicated that HFD downregulated the expression of N-cadherin, Vimentin, matrix metalloproteinase (MMP)2, and MMP9 proteins, and upregulated the expression of E-cadherin protein in GBM cells compared with the control (DMSO) (Figure 2c,d). Collectively, these results showed that HFD significantly inhibited the migration and invasion ability of GBM cells.

### 2.3. Network Pharmacology

**Figure 3:**
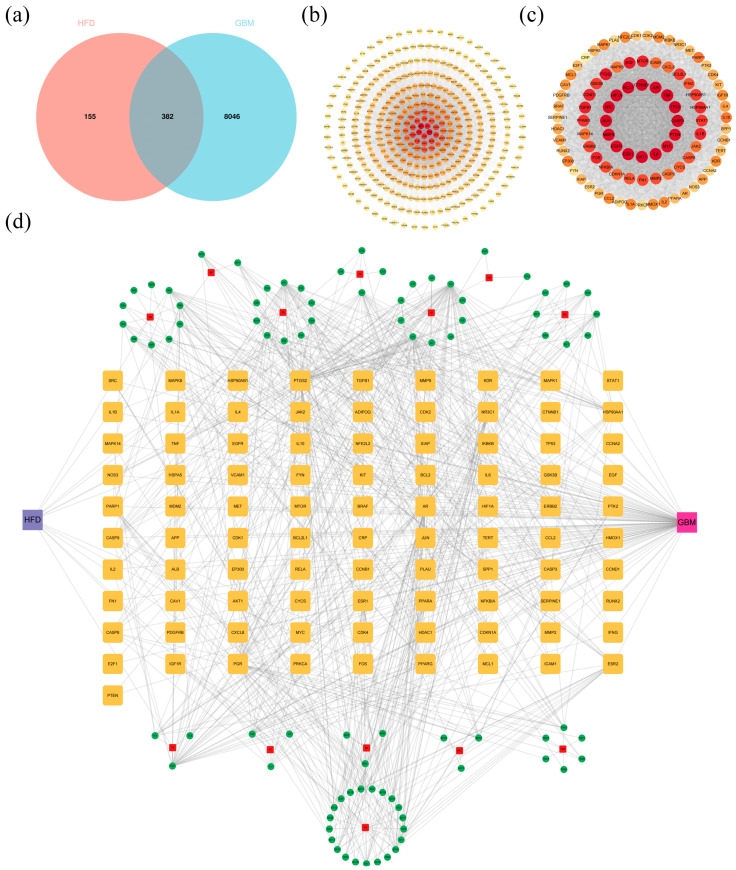
Prediction of HFD in treating GBM by using network pharmacology. (**a**) Venn diagram of HFD drug targets and GBM disease targets. (**b**) Interaction diagram of the PPI network for HFD and GBM intersection targets. (**c**) Interaction diagram of the PPI network for Hub Genes. (**d**) HFD-Chinese medicine–active ingredient–hub gene–GBM network diagram. PPI, protein–protein interaction.

A total of 108 active ingredients were obtained by searching the TCMSP and HERB databases after removing duplicate entries. The number of active targets retrieved from the databases for each TCM was 43 for Typhonii Rhizoma, 168 for *Arum ternatum* Thunb., 147 for *Borneolum syntheticum*, 68 for *Atractylodes lancea* (Thunb.) DC., 6 for Aconiti Radix, 307 for Schizonepetae Herba, 97 for *Santalum album* L., 79 for Arisaematis Rhizoma, 77 for Curcumae Radix, 149 for *Perilla frutescens*, 387 for *Moschus moschiferus*, 325 for *Gastrodia elata*, and 44 for Crotonis Fructus. A total of 537 predicted targets were obtained by summarizing and de-emphasizing all the targets. A total of 8428 GBM-related disease targets were retrieved from the GeneCards database. By taking the intersection of the active ingredient targets of the drug with disease targets, a total of 382 targets of HFD acting on GBM were obtained (Figure 3a). The interactions between the 382 targets were obtained using the STRING database, and the results were imported into Cytoscape 3.9.1 for network topology analysis (Figure 3b). A total of 91 hub genes were screened according to the degree value greater than twice its median, BC value greater than its median, and CC value greater than its median. The color of nodes was set according to the size of degree value. The major targets included AKT1, TP53, TNF, IL6, ALB, SRC, CTNNB1, BCL2, IL1B, and CASP3, suggesting their important roles in the network (Figure 3c).

Ninety-one hub genes were matched to 70 active ingredients, and the network of relationships between the active ingredients and targets was plotted and analyzed using Cytoscape 3.9.1. A total of 191 nodes and 747 relationships were obtained (Figure 3d). The main active ingredients in HFD that were closely related to GBM were quercetin, luteolin, wogonin, morin, and baicalein.

### 2.4. Functional Enrichment of Hub Genes

**Figure 4:**
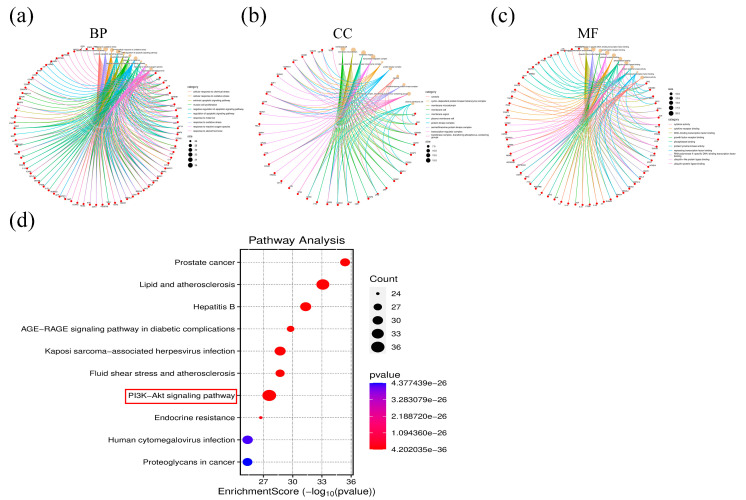
GO and KEGG enrichment analysis of hub genes. (**a**–**c**) Chord plot of GO enrichment results based on hub genes. (**d**) Bubble plot of KEGG enrichment results based on hub genes. GO: Gene Ontology; KEGG: Kyoto Encyclopedia of Genes and Genomes.

GO functional enrichment and KEGG pathway enrichment analyses were performed on these 91 genes to better understand the functions and specific mechanisms of the hub genes. GO functional enrichment analyses showed that in biological processes (BP), hub genes were mainly enriched in the pathways “cellular response to chemical stress”, “response to oxidative stress”, and “cellular response to oxidative stress” (Figure 4a). In cellular components (CCs), these hub genes are mainly located in the “membrane raft”, “membrane microdomain”, and “membrane region” (Figure 4b). Regarding molecular function (MF) analysis, the main items related to the hub gene were “DNA-binding transcription factor binding”, “RNA polymerase II-specific DNA-binding transcription factor binding”, and “growth factor receptor binding” (Figure 4c). KEGG pathway enrichment analysis revealed the hub gene to be closely related to the “PI3K-Akt signaling pathway”, “Lipids and atherosclerosis”, and “Hepatitis B” pathways (Figure 4d).

### 2.5. Identification of 1267 DEGs and 5 Key Modules in GSE209547

**Figure 5:**
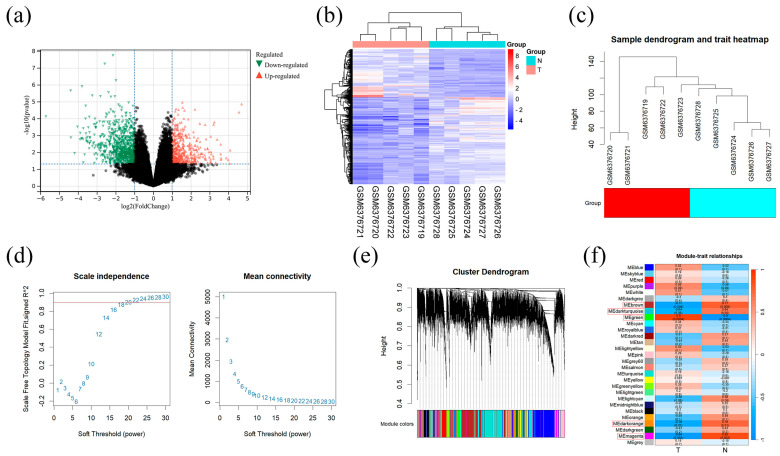
Differential expression analysis of the GBM dataset and WGCNA. (**a**) Volcano plot of DEGs in the GBM dataset. (**b**) Heatmap of DEGs in the GBM dataset. (**c**) Tree clustering diagram in the GBM dataset. (**d**) Scatter plot of the average connectivity and power values. (**e**) Gene clustering after merging similar modules. (**f**) Correlation diagram between gene modules and GBM. DEG, differentially expressed gene; WGCNA, weighted gene co-expression network analysis.

Results of the difference analysis between the GBM sample (T) and normal sample (N) showed a total of 1267 DEGs, of which 451 were upregulated and 816 were downregulated. The results are presented as volcano plots and heatmaps (Figure 5a,b). WGCNA was performed to identify the most relevant gene modules in GBM and normal samples. For this analysis, a soft threshold of 18 was selected based on scale independence and average connectivity, and 29 modules were generated using this threshold. Figure 5c,d, and e show the sample clustering dendrogram, the scatterplot corresponding to the mean connectivity and power values, and the gene clustering dendrogram after merging similar modules, respectively. In addition, the correlation between GBM and gene modules was explored (Figure 5f). As shown in the correlation heatmap, there were five modules with *p* < 0.05, namely brown, dark turquoise, green, dark orange and magenta. The results indicated that these five modules were closely related to GBM. Based on our findings, these five modules were considered as key modules for subsequent analyses.

### 2.6. Identification of 1164 DEGs Using RNA-Seq

**Figure 6:**
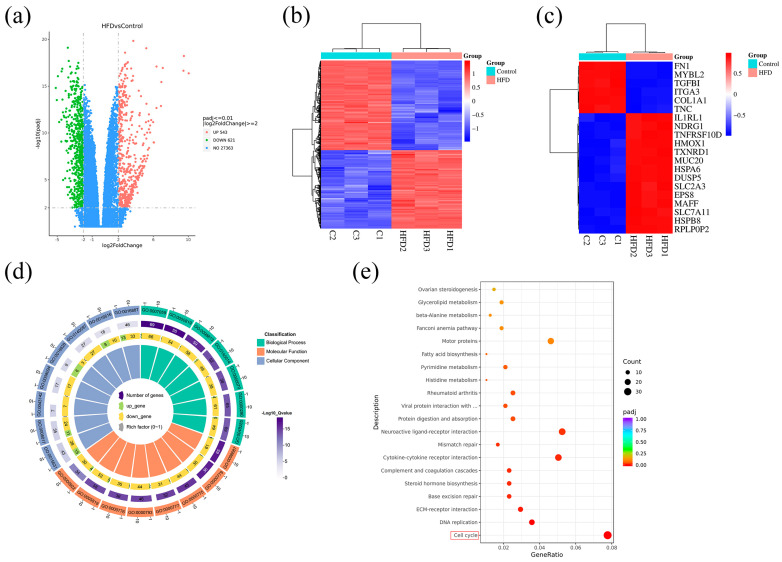
Differential expression analysis of GBM cell transcriptome data. (**a**) Volcano plots of DEGs were compared between the HFD and control groups. (**b**) Heatmaps of DEGs were compared between the HFD and control groups. (**c**) Heatmaps of the top 20 DEGs were compared between the HFD and control groups. (**d**) Circle diagram of GO enrichment results based on DEGs. (**e**) Bubble plot of KEGG enrichment results based on DEGs.

To further explore the potential candidate genes for the treatment of GBM using HFD, transcriptome sequencing was performed on U87 cells from the HFD and control groups. The heatmap and volcano plot showed a total of 1164 differentially expressed genes between the HFD and control groups, of which 543 were upregulated and 621 were downregulated (Figure 6a,b). The top 20 DEGs are shown using the heatmap (Figure 6c). Subsequently, GO and KEGG enrichment analyses were performed on the DEGs, and the GO circle plot showed that the BP-enriched entries were mainly GO:0007059, GO:0000819, and GO:0098813, the CC-enriched entries were mainly GO:0015631, GO:0008017, and GO:0043142, and the MF-enriched entries were mainly GO:0098687, GO:0000779, and GO:0000775 (Figure 6d). The main KEGG-enriched pathways were the cell cycle, DNA replication, and ECM–receptor interaction (Figure 6e).

### 2.7. PLAU and CAV1 Were the Key Genes in HFD for the Treatment of GBM

**Figure 7:**
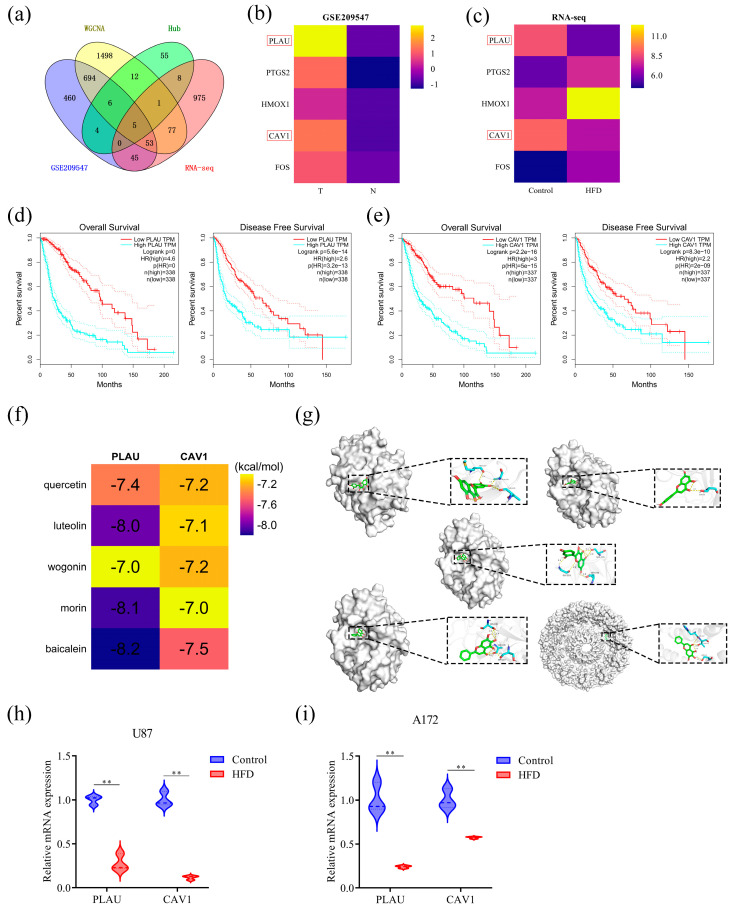
*PLAU* and *CAV1* were the key genes in HFD for the treatment of GBM. (**a**) Wayne diagram after taking the intersection of the following 4 sections of genes: hub genes in network pharmacology, DEGs in GSE209547, genes in the five key modules of WGCNA, and DEGs by RNA-seq. (**b**) Heatmap of intersecting gene expression in GSE209547. (**c**) Heatmap of intersecting genes expression by RNA-seq. (**d**,**e**) Kaplan–Meier survival curve analysis of high and low expression of *PLAU* and *CAV1* in patients with GBM. (**f**) Docking results of the top five absolute binding energies of the main active ingredients after docking with PLAU and CAV1. (**g**) Heatmap of the docking results of the main active ingredients with PLAU and CAV1. (**h**,**i**) Relative mRNA expression of PLAU and CAV1 in U87 and A172 cells, data are presented as mean ± SD, n = 3, ** *p* < 0.01, compared with the DMSO group.

To further identify the key genes for the treatment of GBM using HFD, the results of the following four parts of the analysis were taken based on intersection: hub genes in network pharmacology, DEGs in the GSE209547 dataset, key modular genes in WGCNA, and DEGs in RNA-seq. This yielded a total of five key genes for the treatment of GBM using HFD (Figure 7a). The expression of these five genes in the GSE209547 dataset and by RNA-seq were visualized in the form of heatmaps, which showed that the three genes *PTGS2, HMOX1*, and *FOS* were upregulated in both the GSE209547 dataset and by RNA-seq, and only two genes, *PLAU* and *CAV1*, were upregulated in the GSE209547 dataset. However, in RNA-seq, both genes were downregulated compared with that of the control group (Figure 7b,c). These results suggested that *PLAU* and *CAV1* may be the key genes in HFD for the treatment of GBM. Subsequently, the survival prognosis of these two genes was analyzed using the GEPIA database, and the Kaplan–Meier survival analysis curves showed that patients with low expression of the *PLAU* and *CAV1* had longer overall and disease-free survival versus those with high expression, suggesting that these two genes are closely associated with the survival prognosis of patients with GBM (Figure 7d,e).

### 2.8. Molecular Docking and Validation Using qPCR

The five main active ingredients were each molecularly docked with the two targets, PLAU and CAV1, and the docking results are shown in the heatmap (Figure 7f). The binding energies of docking of PLAU with quercetin, luteolin, wogonin, morin, and baicalein were −7.4 kcal/mol, −8.0 kcal/mol, −7.0 kcal/mol, −8.1 kcal/mol, and −8.2 kcal/mol, respectively, and the binding energies of docking of CAV1 with quercetin, luteolin, wogonin, morin, and baicalein were −7.2 kcal/mol, −7.1 kcal/mol, −7.2 kcal/mol, −7.0 kcal/mol, and −7.5 kcal/mol, respectively. The binding energies for docking were all ≤ −7.0, indicating that the main active ingredients could bind well to both targets. The top five docking results in terms of absolute values of binding energy were selected and visualized using PyMOL; they were quercetin vs. PLAU, luteolin vs. PLAU, morin vs. PLAU, baicalein vs. PLAU, and baicalein vs. CAV1 (Figure 7g).

In addition, mRNA in U87 and A172 cells was extracted for qPCR, and the relative mRNA expression of PLAU and CAV1 in GBM cells was found to be significantly lower than that in control cells (DMSO) after treatment with HFD (Figure 7h,i).

### 2.9. HFD Could Bind Stably to PLAU and CAV1

**Figure 8:**
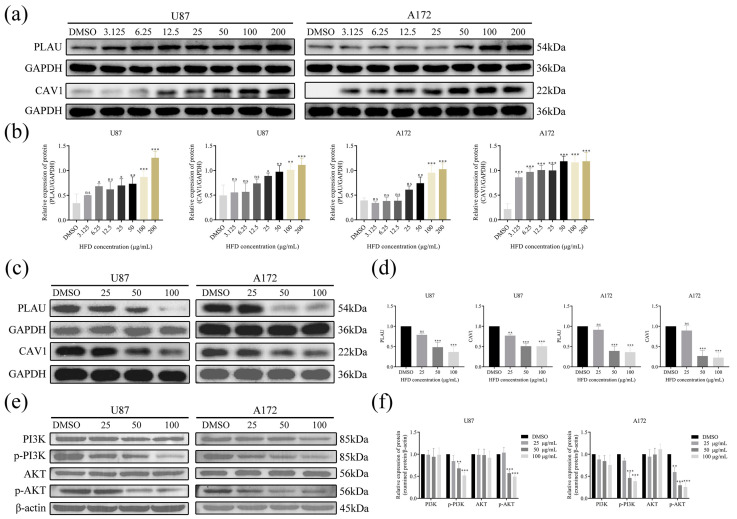
HFD binds stably to and downregulates PLAU and CAV1 protein expression in GBM cells. (**a**,**b**) CETSA to determine the binding efficiency of HFD to PLAU and CAV1 after treatment of U87 and A172 cells with different HFD concentrations. (**c**,**d**) Western blotting for PLAU and CAV1 protein expression after treatment of U87 and A172 cells with different HFD concentrations. (**e**,**f**) Western blotting to determine the expression of PI3K, p-PI3K, AKT, and p-AKT proteins after treating U87 and A172 cells with different HFD concentrations. Data are presented as mean ± SD, n = 3, * *p* < 0.05; ** *p* < 0.01; *** *p* < 0.001, compared with the DMSO group. CETSA, cellular thermal transfer assay; DMSO, dimethyl sulfoxide.

The binding efficiency of HFD with PLAU and CAV1 was further determined using CETSA. The expression of PLAU and CAV1 proteins increased significantly with an increase in HFD concentration compared with that in the control group, indicating that the binding of HFD with PLAU and CAV1 had good thermal stability (Figure 8a,b).

### 2.10. HFD Downregulated the Expression of PLAU and CAV1 for Modulating the PI3K/AKT Signaling Pathway

Changes in the expression of PLAU and CAV1 proteins in GBM cells were determined using Western blotting. PLAU and CAV1 protein expression in GBM cells from the HFD-treated group was significantly reduced compared with that in control cells (Figure 8c,d). In addition, after HFD treatment, there was no significant change in PI3K and AKT protein expression in GBM cells, whereas p-PI3K and p-AKT protein expression was significantly downregulated, suggesting that HFD may exert its anti-GBM effect by regulating the PI3K/AKT signaling pathway (Figure 8e,f).

### 2.11. HFD Significantly Inhibited GBM Growth In Vivo and Exhibited No Apparent Toxicity

**Figure 9:**
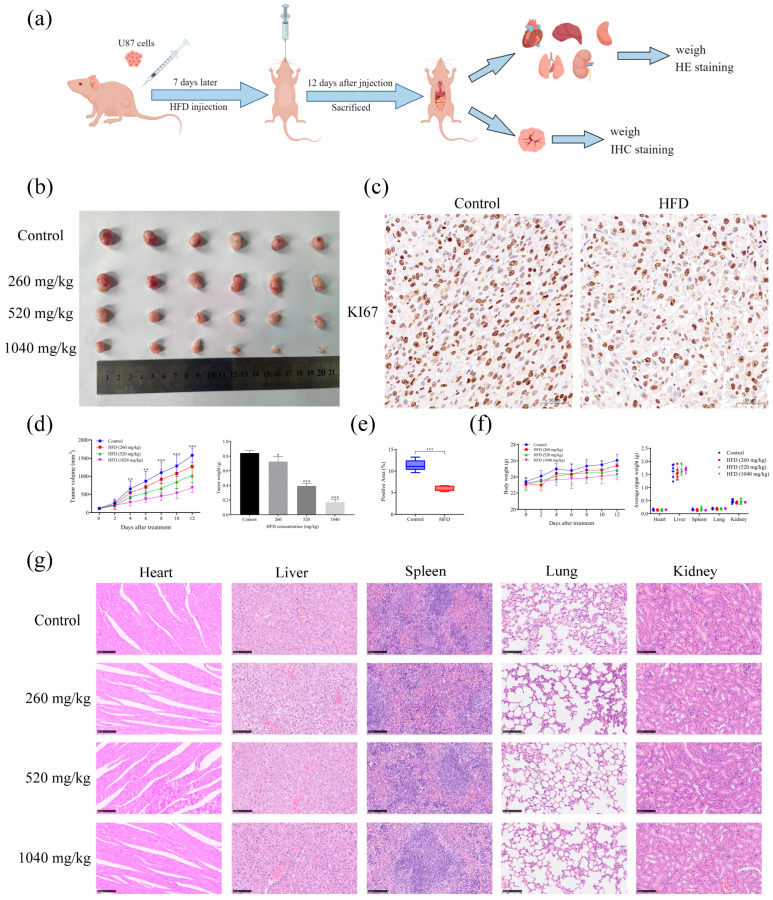
HFD inhibited GBM growth in vivo. (**a**) Flowchart of the establishment of a subcutaneous tumor graft model. (**b**) Tumor images of mice. (**c**) Tumor volume and weight statistics in mice. (**d**,**e**) Positive KI67 expression in tumor tissues determined using IHC. (**f**) Weights and weight statistics of each internal organ of mice. (**g**) HE staining of tissues of various internal organs of mice. Data are presented as mean ± SD, n = 5, * *p* < 0.05; ** *p* < 0.01; *** *p* < 0.001, compared with the control group. HE, hematoxylin and eosin; IHC, immunohistochemical.

U87 cells were inoculated subcutaneously into BALB/c nude mice. A nude mouse subcutaneous graft tumor model was established to assess the in vivo efficacy of HFD (Figure 9a). HFD significantly inhibited tumor growth (Figure 9b,d) compared with that in the control group. Subsequently, the tumor tissues of mice were taken for immunohistochemical staining to determine KI67 expression in vivo. KI67 expression in the HFD-treated group (1040 mg/kg) was significantly lower than that in the control group (Figure 9c,e). In addition, results from HE staining showed that HFD had no obvious in vivo toxicity (Figure 9f,g). These results suggested the anti-GBM effect of HFD and its potential as a candidate drug to treat GBM.

## 3. Materials and Methods

### 3.1. Materials, Cells, and Experimental Animals

Human GBM cell lines (U251, U87, and A172) were purchased from the Cell Resource Center of Shanghai Institutes for Biological Sciences, Chinese Academy of Sciences (SIBS, CAS) and preserved at the Guizhou Natural Products Research Center (Guiyang, China).

Liao’s Huafengdan (HFD, approval number Z20026460) was manufactured by Guizhou Wansheng Pharmaceutical Limited Liability Company (Zunyi, Guizhou Province, China), and it was prepared into a master batch of 100 mg/mL with dimethyl sulfoxide (DMSO; Guodinchangsheng Biotechnology Co., Ltd., Beijing, China), which was gradient-diluted into the corresponding working solution during the experiment. Tetramethyl azole blue (MTT; M8180) was purchased from Beijing Solebaum Biotechnology Co., Ltd. (Beijing, China); an EDU Cell Proliferation Detection Kit (100–121) and Cell Cycle Detection Kit (100–107) were purchased from Albatross Bioscience Co., Ltd. (Guangzhou, Guangdong Province, China); SweScript All-in-One RT SuperMix for qPCR (One-Step gDNA Remover) (G3337) was acquired from Wuhan Xavier Biotechnology Co., Ltd. (Wuhan, Hubei Province, China); Matrigel gel (354234) was obtained from Corning Incorporated (Corning, New York, NY, USA); the stripping solution (FD0050) was purchased from Hangzhou Fode Biotechnology Co., Ltd. (Hangzhou, Zhejiang Province, China); Antibodies against proliferating cell nuclear antigen (PCNA; #13110), Cyclin A2 (#91500), E-cadherin (#3195), Vimentin (#5741), N-cadherin (#13116), MMP2 (#4022), MMP9 (#13667), and β-actin (#4967) were acquired from CST (Shanghai, China); Cyclin-dependent kinase 2 (CDK2; ET1602-6) and protein kinase B (AKT; ET1609-51) were acquired from Hangzhou Hua’an Bio-technology Co., Ltd. (Hangzhou, Zhejiang Province, China); phosphorylated (p)-PI3K (bs-3332R) was obtained from Beijing Boao Sen Biotechnology Co. (Beijing, China); phosphatidylinositol 3-kinase (PI3K; 60225-1-Ig), Urokinase-type plasminogen activator (PLAU; 17968-1-AP), and Caveolin-1 (CAV1; 16447-1-AP) were purchased from Wuhan Sanying Biotechnology Co., Ltd. (Wuhan, Hubei Province, China); phosphorylated (p)-AKT (GB150002) and KI67 (GB121141) were acquired from Wuhan Xavier Bio-technology Ltd. (Wuhan, Hubei Province, China); GAPDH (ET1601-4) was obtained from Guizhou Huayuan Biotechnology Co. (Guiyang, Guizhou, China).

The experimental animals used in this study were SPF-grade male BALB/c nude mice, aged 5–6 weeks and weighing 22–24 g. The mice were provided by Hangzhou Ziyuan Laboratory Animal Science and Technology Co., Ltd. (Hangzhou, China) with an animal production license (Certificate No.: SCXK (Zhejiang) 2019-0004). The animal experiments were approved by the Ethics Committee of Guizhou Medical University and were conducted in accordance with the Guidelines for Ethical Review of Laboratory Animal Welfare in China (Approval No.: 2403626).

### 3.2. Cell Culture

U251, U87, and A172 cells were cultured in Dulbecco’s Modified Eagle Medium (DMEM; Gibco, New York, NY, USA) containing 10% fetal bovine serum (FBS; Sijiqing Biotechnology Co., Ltd., Hangzhou, Zhejiang Province, China) and 1% penicillin/streptomycin (Solarbio, Beijing, China) at 37 °C in an incubator flushed with 5% CO_2_.

### 3.3. MTT Assay

GBM cells in the logarithmic growth phase were inoculated into 96-well culture plates at a density of 5 × 10^3^ per well. Once the cells had attached to the walls of the plate, different concentrations of HFD (6.25 μg/mL, 12.5 μg/mL, 25 μg/mL, 50 μg/mL, and 100 μg/mL) were added. An equal volume of dimethylsulfoxide (DMSO) added to cells served as the control group. All cells were incubated for 24 and 48 h. Next, 20 μL of MTT solution was added to cells and incubated for 4 h away from light, and the supernatant was discarded. Next, 20 μL of MTT solution was added while avoiding light, and the cells were further incubated for 4 h. The supernatant was discarded and 150 μL of DMSO solution was added to each well. The plate was kept on an oscillator for 10 min and the absorbance at 490 nm was determined using an enzyme marker. The proliferation rate of each group of cells was statistically analyzed.

### 3.4. 5-Ethynyl-2′-Deoxyuridine (EdU) Staining Assay

U87 and A172 cells were inoculated into 12-well plates at a density of 2 × 10^4^ cells per well. Once the cells had adhered to the walls of the plate, they were treated with different concentrations of HFD (0 μg/mL, 25 μg/mL, 50 μg/mL, and 100 μg/mL). After treatment for 24 h, the medium was discarded and the cells were rinsed twice with phosphate-buffered saline (PBS) and treated with EdU reagent for 2 h per the manufacturer’s instructions. The cells were fixed with 4% paraformaldehyde for 10 min and treated with 0.2% Triton X-100 for 10 min. The nuclei were stained with Hoechst 33342 and fluorescence was determined using an inverted fluorescence microscope (DMI8, Leica, Hesse, Germany).

### 3.5. Cell Cycle Assay

U87 and A172 cells were inoculated in 6-well plates at a density of 2 × 10^4^ cells per well. Once the cells had adhered to the walls of the plate, different concentrations of HFD (0 μg/mL, 25 μg/mL, 50 μg/mL, and 100 μg/mL) were added to cells. The cells were digested and collected after 24 h of treatment, and 300 µL of PBS was added to the sample taken from each group to prepare a cell suspension. Next, 700 µL of precooled anhydrous ethanol (70% by mass) was slowly added to cells and fixed overnight in a refrigerator at 4 °C. Ethanol was removed by centrifugation, DNA staining solution was added, and the cells were incubated for 30 min at room temperature and away from light. Cell cycle distribution was analyzed using a NovoCyte 2040R flow cytometer (BioTek, Winooski, VT, USA).

### 3.6. Cell Apoptosis Analysis

After treating GBM cells with different concentrations of HFD (0 μg/mL, 25 μg/mL, 50 μg/mL, and 100 μg/mL) for 24 h, the medium was collected into a centrifuge tube. The cells were rinsed once with precooled PBS and digested with EDTA-free trypsin. The cells were collected, and the medium collected in the previous step was added, mixed homogeneously, and transferred to a new centrifuge tube. After centrifuging at 1000 rpm for 5 min, the supernatant was discarded, and the collected cells were rinsed twice with precooled PBS. The cells were resuspended in 1× binding buffer, and 5 µL of Annexin V-FITC and 10 µL of PI were added. Flow cytometry was performed after incubating the cells for 30 min at room temperature and away from light. The apoptosis rate of cells in each group was statistically analyzed.

### 3.7. Transwell Assay

For the cell migration assay, GBM cells were digested and resuspended in a serum-free medium. Next, the cells were counted and inoculated into the upper chamber of a 24-well plate at a density of 2 × 10^4^ cells per well. Then, 700 µL of DMEM containing 10% FBS was added to the lower chamber and the cells were further cultured for 24 h in the incubator. For the invasion assay, a layer of Matrigel adhesive was applied evenly to the upper surface of the PET film of the chambers. The chambers were gently placed into the wells of the 24-well plate, left undisturbed for about 3 h at 37 °C, removed, and allowed to air-dry overnight on an ultra-clean bench. The 24-well plate was removed, the medium was discarded, and the cells were fixed with 4% paraformaldehyde for 30 min and stained with 0.5% crystal violet for 30 min. The cells were photographed using an inverted microscope (Olympus; Beijing, China), and the extent of cell migration and invasion in each group was determined. Cells were photographed using the Olympus inverted microscope (Beijing, China) and the number of cells that had migrated and invaded in each group was counted.

### 3.8. Subcutaneous Tumor Formation in Nude Mice

U87 cells in the logarithmic growth phase were resuspended in NaCl at a density of 1 × 10^7^ cells/mL. The cells were then subcutaneously inoculated on the back of mice on the right side. Inoculation was considered successful when a small subcutaneous bulge was observed. When the tumor volume of the mice increased to approximately 100 mm^3^, the mice were randomly divided into a control group or a treatment group. Mice in the treatment group were gavaged with HFD at doses of 260 mg/kg, 520 mg/kg, and 1040 mg/kg, respectively, whereas those in the control group were gavaged with the same dose of saline. The drugs were administered daily, and the body weights (g) and tumor volumes (a: length, b: width; tumor volume = a × b^2^ × 0.5) of the mice were recorded every 2 days. After 12 days of drug administration, the mice were executed and their hearts, livers, spleens, lungs, kidneys, and tumors were weighed.

### 3.9. Immunohistochemical (IHC) Staining

The tumor tissue samples (control group and HFD 1040 mg/kg group) were sliced, and the slices were soaked in different gradient solutions of xylene and ethanol for dewaxing and rehydration. Antigen repair and recovery were performed using sodium citrate, and endogenous peroxidase activity was blocked using 3% H_2_O_2_. Tissue samples were incubated overnight with KI67 (dilution 1:400) at 4 °C after blocking with 3% bovine serum albumin (BSA) at room temperature for 30 min. After the unbound antibody was washed with PBS, the glioma tissues were incubated with the secondary antibody and stained with DAB and hematoxylin. Next, the sections were dehydrated and sealed, dried in a fume hood, and observed using an inverted microscope to acquire images. The percentage of KI67-positive area was counted using ImageJ 1.8.0.

### 3.10. Hematoxylin and Eosin (HE) Staining

The tissue sections of each organ were treated with xylene and ethanol solutions for gradient dewaxing and hydration, stained with hematoxylin, differentiated with alcoholic hydrochloric acid, and stained with eosin after re-blueing with ammonia. The sections were then dehydrated and clarified using solvent gradients, transparentized using ethanol and xylene solution, sealed with neutral gum, dried in the fume cupboard, and observed using an inverted microscope to acquire images.

### 3.11. Network Pharmacology Analysis

The TCMSP (https://old.tcmsp-e.com/tcmsp.php, (accessed on 10 July 2023)) and HERB (http://herb.ac.cn/, (accessed on 18 July 2023)) databases were searched to obtain the active ingredients in the 13 herbal medicines (Arisaematis Rhizoma, *Arum ternatum* Thunb., Typhonii Rhizoma, Aconiti Radix, *Gastrodia elata*, *Moschus moschiferus*, *Santalum album* L., *Borneolum syntheticum*, *Atractylodes lancea* (Thunb.) DC., *Perilla frutescens*, Schizonepetae Herba, Crotonis Fructus, and Curcumae Radix) in HFD. The screening criteria in the TCMSP database were based on the oral bioavailability (OB) of the compounds being >30% and the drug-like properties (DL) being >0.18, whereas the ingredients obtained from the HERB database were screened using the SwissADME (http://www.swissadme.ch/index.php, (accessed on 13 March 2025)) database using the following conditions: GI absorption was high; at least three of Lipinski, Ghose, Veber, Egan, and Muegge were “YES”; and MW was <500. The obtained active ingredients were integrated using Microsoft Excel 2019, and after de-emphasis, the candidate active ingredients were standardized. Next, the relevant targets of action of the active ingredients were collected from the database, and if there were no active ingredients with relevant targets, their SMILES numbers were imported into the SwissTargetPrediction (http://www.swisstargetprediction.ch/, (accessed on 13 March 2025)) database, and their targets of action were screened with probability > 0. The targets obtained from the databases were combined, and the targets of all active ingredients of HFD were obtained after de-emphasis. Using “glioblastoma (GBM)” as the search term, GBM-related targets were retrieved from the GeneCards database (https://www.genecards.org/, (accessed on 11 July 2023)) and summarized and organized into GBM-related disease targets using Microsoft Excel 2019. The intersection of active ingredient targets of HFD and disease targets of GBM was taken to obtain the potential therapeutic targets of HFD for GBM. The intersected targets were imported into the STRING database (https://cn.string-db.org/, (accessed on 19 July 2023)), the species was selected as “*Homo Sapiens*”, the interactions between the targets were obtained, a protein–protein interaction (PPI) network was constructed, and the .TSV format file was saved. The .TSV file was imported into Cytoscape 3.9.1 and the built-in data-analysis function “Network Analyzer” was used to calculate the topological parameters of each target in the network. Nodes were evaluated using two times the median node degree of freedom (degree), the median of intermediate centrality (BC), and the median of near centrality (CC). Hub genes were defined as points with a degree value > 2 times the median, and with both BC and CC values > the median. The hub genes and corresponding active ingredients were sorted out using Microsoft Excel 2019, and data were imported into Cytoscape 3.9.1 to construct the HFD—herbal medicine–active ingredient–hub gene–GBM network diagram. The network relationship between the nodes was analyzed using the CytoNCA plug-in to screen out the main active ingredients.

### 3.12. Gene Ontology (GO) and Kyoto Encyclopedia of Genes and Genomes (KEGG) Enrichment Analysis

To determine the biological functions and specific mechanisms of Hub genes, GO functional enrichment analysis and KEGG pathway enrichment analysis of hub genes were performed using the DAVID database (https://david.ncifcrf.gov/, (accessed on 19 July 2023)). *p* < 0.05 was considered as significant enrichment. In addition, the enrichment results were visualized using the R packages “ggplot2” and “circlize” (R 4.4.2). Results are presented using chordal graphs and bubble plots.

### 3.13. Microarray Data Collection and Analysis of Differentially Expressed Genes (DEGs)

The original expression profiles of GBM tissues (T) and normal tissues (N) were downloaded from the GEO database (https://www.ncbi.nlm.nih.gov/geo/, (accessed on 12 July 2023)) and the GSE209547 dataset (platform No. GPL16043, including 5 GBM samples and 5 normal samples). The GBM dataset (GSE209547) was analyzed for DEGs using the “limma” package in R software (Version: 4.4.2), with a screening threshold of *p* < 0.05 and |log (fold change)| > 1. The results were visualized using R packages “pheatmap” and “ggplot2”, and volcano plots and heatmaps were drawn.

### 3.14. Weighted Gene Co-Expression Network Analysis (WGCNA) and Identification of Key Modular Genes

A scale-free co-expression gene network for GSE209547 was constructed using the WGCNA package, and the variance of each gene in the data was calculated and the top 50% of genes were filtered. The “good Samples Genes” function was used to check the dataset for missing terms, terms with weights below the threshold, and zero-variance genes, which returned a list of samples and genes with the maximum missing values or low weight value criteria. Moreover, the soft threshold of the dataset was set to 18 as the weight value for this experiment. The correlation between the module feature matrix and the sample information matrix was calculated and the above correlation matrix and *p* value were visualized using the R package “labeledHeatmap”. The modules whose positive and negative correlations were significant at *p* < 0.05 were selected as the key module genes in the GBM dataset.

### 3.15. Transcriptome Sequencing and Analysis of DEGs

After treatment of U87 cells with HFD (100 μg/mL), cell precipitates from the HFD and control groups were collected, and total cellular RNA was extracted using TRIzol reagent according to the manufacturer’s instructions. RNA integrity and the total amount of RNA were determined using an Agilent 2100 bioanalyzer (Agilent Technologies, Santa Clara, CA, USA). mRNA was subsequently enriched for cDNA synthesis using Oligo(dT) beads. cDNA was screened for amplification using polymerase chain reaction (PCR) and AMPure XP beads (Beckman Coulter, Brea, CA, USA), and the PCR products were purified using AMPure XP beads to generate the final libraries. Illumina sequencing was performed after quality control of the library. Transcriptome sequencing and analysis were performed by Shanghai Novogene Corporation (Shanghai, China). Transcriptome data were analyzed using the “limma” package in R software, and the cutoff values were *p* adjust value < 0.01 and |log2 fold change| > 2 to obtain DEGs between the Control and HFD groups. Subsequently, the R packages “pheatmap” and “ggplot2” were used to visualize the DEGs and draw volcano plots and heatmaps.

### 3.16. Identification of Key Genes and Analysis of Survival Prognosis

The results of the analysis of the following four parts were taken as intersections to identify the key genes of HFD in treating GBM—the hub genes in the network pharmacology part, DEGs in the GSE209547 dataset, key modular genes in the WGCNA, and DEGs from RNA-seq—and the results of the intersections were visualized using a Wayne diagram. The expression of the intersected genes in the GSE209547 dataset and RNA-seq were each analyzed, and the expression was visualized using a heatmap to ultimately identify the key genes for the treatment of GBM by HFD. These key genes were subjected to survival prognostic analysis using the GEPIA (http://gepia.cancer-pku.cn/detail.php###, (accessed on 13 March 2025)) database and the results were visualized.

### 3.17. Molecular Docking of Key Genes with Major Active Ingredients

The 2D structures of the main active ingredients were downloaded from the PubChem database (https://pubchem.ncbi.nlm.nih.gov/, (accessed on 12 August 2023)), imported into ChemBio3DUltra 14.0 software, and subjected to energy minimization. The files were saved in the mol2 format. The 3D structures of the key genes were downloaded from the PDB (https://www.rcsb.org/, (accessed on 25 September 2023)) database, imported into PyMOL 2.6.0 software, subjected to dewatering and de-residue ligand processing, and saved as .Pdb files. Subsequently, the structures of the main active ingredients and key genes were imported into AutoDockTools 1.5.6 software for hydrogenation and identification of ligands and receptors and then saved in the .pdbqt format. Active pockets for the docking of the major active ingredients and key genes were searched and saved using AutoDockTools, followed by molecular docking by AutoDock Vina. Docking results were visualized using PyMOL 2.6.0.

### 3.18. Real-Time Fluorescence Quantitative PCR (qPCR)

After treating U87 and A172 cells with HFD (100 μg/mL), the cell precipitates were collected from the HFD and control groups and were each lysed with Trizol. Total cellular RNA was extracted according to the manufacturer’s instructions, and RNA concentration and purity were determined using a NanoDrop 2000 Micro Nucleic Acid Quantifier (Wuhan servicebio technology CO., Ltd., Wuhan, Hubei Province, China). RNA was reverse-transcribed to cDNA using the One-Tube One-Step Genomic DNA Removal and Reverse Transcription kit according to the manufacturer’s instructions. qPCR was subsequently performed using the QuantStudio 5 Real-Time Fluorescent Quantitative PCR system (Bio-Rad, Hercules, CA, USA) with SYBR Green as the detection fluorophore. To standardize gene expression of GAPDH, the 2^−ΔΔCt^ method was used to determine relative RNA expression. Primer sequences are listed in Table 1.

### 3.19. Cellular Thermal Transfer Assay (CETSA)

U87 and A172 cells were spread into large dishes. After the cells had adhered to the walls of the dish, they were treated with different concentrations of HFD (0 μg/mL, 3.125 μg/mL, 6.25 μg/mL, 12.5 μg/mL, 25 μg/mL, 50 μg/mL, 100 μg/mL, and 200 μg/mL) for 2 h. Cells were collected separately and treated in a metal bath at 52 °C for 3 min, and then cooled to room temperature and set aside. An Eppendorf tube with the cell suspension was put on the floating plate and immersed in liquid nitrogen for 1 min to freeze and then immediately put into a water bath at 37 °C to melt. This process was performed 3 times. After centrifugation at 12,000× *g* for 5 min at 4 °C, the supernatant was collected. The binding efficiency of HFD to plasminogen activator urokinase (PLAU) and caveolin-1 (CAV1) proteins was determined using Western blotting.

### 3.20. Western Blotting

Total proteins were extracted from GBM cells and subjected to sodium dodecyl sulfate–polyacrylamide gel electrophoresis. The separated proteins were transferred from the gel to a polyvinylidene fluoride membrane. After sealing the membrane with 5% BSA, the membrane was incubated overnight at 4 °C with the primary antibody. Next, the membrane was washed 3 times with TBST and then incubated with the secondary antibody at room temperature for 2 h. The membrane was visualized using ECL Extreme Ultrasensitive Luminescent Solution using a gel imaging system (U: Genius 3, Hong Kong Gene Co., Ltd., Hong Kong, China). The gray value of protein bands was determined using ImageJ.

### 3.21. Statistical Analysis

IBM SPSS 23.0 and GraphPad Prism 9.5 were used for statistical analysis. Results are expressed as mean ± standard deviation. The normality of each dataset was assessed using the Shapiro–Wilk method to confirm that the criteria for normal distribution were met. In addition, the Levene method was used to test for chi-square distribution. Subsequently, when the normal and chi-square distribution were satisfied, Student’s *t*-test was used to compare differences between 2 groups, while one-way analysis of variance was used to compare differences between multiple groups. *p* < 0.05 was considered statistically significant.

## 4. Discussion

GBM is a life-threatening glioma, leading to an overall poor prognosis for patients due to its high degree of malignancy and aggressiveness [17,18]. Previous studies have shown the significant anti-GBM effects of HFD both in vitro and in vivo; however, the underlying molecular mechanisms are unclear. One of the most fundamental properties of tumor cells is their ability to proliferate indefinitely, a property that stems from the dysfunction of cell cycle–regulatory genes [19]. Inhibition of the cell cycle, especially the S-phase, has crucial antitumor implications. For example, benzimidazole impedes the progression of human GBM by blocking the cell cycle [20], whereas cordycepin inhibits the expression of cyclin A2 and CDK2, leading to the accumulation of cells in the S-phase [21]. In this study, MTT and EdU assays were used to determine the effect of HFD on GBM cell proliferation. HFD could significantly inhibit the proliferative capacity of GBM cells. In addition, Western blotting showed that HFD could reduce PCNA protein expression in GBM cells. The effects of HFD on the GBM cell cycle and apoptosis were evaluated using flow cytometry and Western blotting. HFD decreased the expression of cyclin A2 and CDK2 proteins, leading to the blocking of the S-phase in GBM cells; and downregulated the expression of Bcl2 protein and upregulated that of Bax protein, eventually leading to apoptosis. Moreover, our findings showed that HFD could significantly inhibit the growth of GBM in vivo; immunohistochemical staining revealed that HFD could reduce KI67 expression in GBM tissues; and HE staining indicated no obvious HFD-induced toxicity. As a first-line chemotherapeutic agent for GBM, temozolomide carries inherent risks of hepatorenal toxicity in clinical practice. In contrast, animal studies have demonstrated that HFD exhibits no significant histopathological damage in liver and kidney tissues based on HE staining findings. This distinct safety profile suggests HFD may offer superior hepatorenal protective advantages in GBM treatment strategies.

Epithelial–mesenchymal transition (EMT) is associated with various aspects of cancer malignancy, including tumor invasion, metastasis formation, and drug resistance. During EMT, cancer cells progressively lose epithelial cell-to-cell adhesion and acquire a mesenchymal phenotype as well as migration and invasion capabilities [22,23,24]. The main challenge that makes it difficult to treat GBM is its characteristic of rapid migration and diffuse tissue infiltration [25]. EMT is considered the main pathway that affects the invasion and metastasis of GBM cells [26]. Representative epithelial–mesenchymal marker proteins reflect this biological process. The Transwell assay in this study showed that HFD inhibited the migration and invasion of GBM cells. Furthermore, Western blotting revealed that HFD could upregulate E-cadherin protein expression and downregulate the expression of N-cadherin, Vimentin, MMP2, and MMP9 proteins, suggesting the ability of the compound to inhibit the migration and invasion of GBM cells by suppressing EMT.

PLAU, also known as urokinase-type plasminogen activator, exerts several biological effects in various physiological and pathological processes such as keratinocyte proliferation [27], airway inflammation [28] and rheumatoid arthritis [29]. PLAU plays a role in tumor progression by promoting tumor cell proliferation and also promoting cell migration, invasion, and EMT [30,31,32]. A study has reported that aberrant PLAU expression is associated with glioma progression and poor prognosis in patients with glioma [33]. Another study has reported that upregulation of PLAU can promote the migration of GBM cells [34]. Collectively, these findings suggest the important role of PLAU in the onset and development of GBM. CAV1 is a regulatory protein located in the fossa region of the cell membrane. It plays a crucial role in the formation of plasma membrane invaginations known as caveolae [35]. In the fovea, CAV1 interacts with several signaling molecules, including G protein–coupled receptors, tyrosine kinases, and GTPases. Several studies have shown that CAV1 overexpression is associated with cancer progression, angiogenesis, and metastasis [36,37]. Several studies have reported that CAV1 can alter the molecular basis of the pathobiology of brain tumors, especially the malignant glial subtypes [38]. Wang [39] et al. found that CAV1 enhances EMT by mediating the activation of PAI-1 and its correlation with immune infiltration, thereby promoting glioma proliferation and metastasis. The findings from these studies suggest that CAV1 may be a potential therapeutic target and prognostic marker for GBM.

The targets of action of HFD in treating GBM and molecular mechanisms were analyzed in this study by integrating network pharmacology, bioinformatics, and transcriptomics. PLAU and CAV1 were determined to be the key targets of HFD in treating GBM, and the related signaling pathways were mainly the PI3K/AKT signaling pathway and the cell cycle. Survival prognostic analysis showed that the low expression of PLAU and CAV1 was positively correlated with good prognosis of GBM. Furthermore, the main active ingredients in HFD responsible for its effect were quercetin, luteolin, wogonin, morin, and baicalin. Next, the binding of the main active ingredients to key genes was simulated using molecular docking. These five main active ingredients exhibited good binding ability to two key genes. Findings from qPCR showed that HFD could significantly downregulate the relative mRNA expression of PLAU and CAV1 in GBM cells. Results from Western blotting indicated that HFD could significantly downregulate the protein expression of PLAU and CAV1 in GBM cells. Furthermore, HFD downregulated the expression of p-PI3K and p-AKT, suggesting that it may exert its anti-GBM effect by regulating the PI3K/AKT signaling pathway.

## 5. Conclusions

Our findings demonstrate that HFD inhibits the growth, migration, and invasion of GBM by acting on the dual targets of PLAU and CAV1 and modulating the PI3K/AKT signaling pathway. This crucial finding indicates the potential of HFD in treating GBM in a clinical setting.

## 6. Patents

The manuscript contains a patent arising from patent application NO. CN202410507099.8.

## Figures and Tables

**Table 1:** Primer sequences used in this study.

Gene	Primer	Primer Sequence
*PLAU*	Forward Reverse	5′-AACGACATTGCCTTGCTGAAGAT-3′ 5′-GTGACTTCAGAGCCGTAGTAGTG-3′
*CAV1*	Forward Reverse	5′-GACCCTAAACACCTCAACGATGA-3′ 5′-CCAGATGTGCAGGAAAGAGAGAA-3′
*GAPDH*	Forward Reverse	5′-GGAAGCTTGTCATCAATGGAAATC-3′ 5′-TGATGACCCTTTTGGCTCCC-3′

## Data Availability

The data supporting the findings of this study are available from the corresponding author upon reasonable request.
