# Correction: Lin et al. Huafengdan Inhibits Glioblastoma Cell Growth and Mobility by Acting on PLAU and CAV1 Targets. *Pharmaceuticals* 2025, *18*, 428

**DOI:** 10.3390/ph18060815

**Published:** 2025-05-29

**Authors:** Dengxiao Lin, Wenfeng Yu, Jia Yu, Sha Cheng, Yu Song, Xiaoqing Wan, Yingjiang Xu, Heng Luo, Baofei Sun

**Affiliations:** 1 Key Laboratory of Human Brain Bank for Functions and Diseases, Department of Education of Guizhou Province, College of Basic Medical, Guizhou Medical University, Guiyang 561113, China; lindengxiao@163.com (D.L.); wenfengyu@gmc.edu.cn (W.Y.); 2 State Key Laboratory of Discovery and Utilization of Functional Components in Traditional Chinese Medicine, Guizhou Medical University, Guiyang 550014, China; yujia@gzcnp.cn (J.Y.); chengsha@gzcnp.cn (S.C.); 3 Natural Products Research Center of Guizhou Province, Guizhou Medical University, Guiyang 550014, China; 4 Guizhou Wansheng Pharmaceutical Co., Ltd., Zunyi 563005, China; 18323539745@163.com (Y.S.); 13985198830@163.com (X.W.); 18781726591@163.com (Y.X.)

## Error in Figure 1

In the original publication [1], there was a mistake in Figure 1f as published. During a post-publication review, we identified an inadvertent error in the Western blot panels of Cyclin A2 and GAPDH in Figure 1f. Specifically, the lanes for Cyclin A2 and GAPDH in the U87 cell line were mistakenly labeled as originating from the A172 cell line, and vice versa. This error occurred during figure assembly and does not affect the experimental results, statistical analysis, or conclusions of the study. The corrected Figure 1 appears below. We state that the scientific conclusions are unaffected. This correction was approved by the Academic Editor. The original publication has also been updated.


